# Long and His Discovery

**Published:** 1908-11

**Authors:** I. H. Goss

**Affiliations:** Athens, Ga.


### Journal-Record of Medicine

Successor to Atlanta Medical and Surgical Journal, Established 1855, and Southern Medical Record, Established 1870.

**OWNED BY THE ATLANTA MEDICAL JOURNAL CO.**

**Published Monthly.**

*Official Organ Fulton County Medical Society, State Examining Board, Presbyterian Hospital, Etc.*

EDGAR G. BALLENGER, M. D., Editor.
BERNARD WOLFF, M. D., Supervising Editor.
A. W. STIRLING, M. D., C. M., D. P. H., J. S. HURT, B. Ph., M. D., Associate Editors
E. W. ALLEN, Business Manager.

COLLABORATORS
DR. W. F. WESTMORLAND, General Surgery.
F. W. McRAE, M. D., Abdominal Surgery.
H. F. HARRIS, M. D., Pathology and Bacteriology.
E. B. BLOCK, M. D., Diseases of the Nervous System.
MICHEL HOKE, M. D., Orthopedic Surgery.
CYRUS W. STRICKLER, M. D., Legal Medicine and Medical Legislation.
E. C. DAVIS, A. B., M. D., Obstetrics.
E. G. JONES, A. B., M. D., Gynecology.
R. T. DORSEY, Jr., B. S. M. D., Medicine.
L. M. GAINES, A. B., M. D., Internal Medicine.
J. N. LeCONTE, M. D., Diseases of the Stomach and Intestines
L. B. CLARKE, M. D., Pediatrics.
EDGAR PAULIN, M. D., Opsonic Medicine.
R. R. DALY, M. D., Medical Society.
A. W. STIRLING, M. D., Etc., Diseases of the Eye, Ear, Nose and Throat.
BERNARD WOLFF, M. D., Diseases of the Skin.
E. G. BALLENGER, M. D., Diseases of the Genito Urinary Organs.

VOL. X. NOVEMBER, 1908. No. 8

### LONG AND HIS DISCOVERY.

BY I. H. GOSS, M. D., ATHENS, GA.

We have been taught that medical operations have been tempered by forms of anaesthesia "since the days whereof the memory of man runneth not to the contrary." The suggestion has been made that the "deep sleep" that the Creator "caused to fall upon Adam" was the germ idea of anaesthesia. There are traditions that the Assyrians employed digital compression of the carotid arteries to produce anaesthesia; also that the Egyptians used Indian hemp and the juice of the poppy to cause drowsiness before surgical operations.

The Odyssey informs us that a "sorrow easing drug" was given by Helen to Ulysses. The younger Pliny describes the use of mandragora as a narcotic, and Galen speaks of its power to paralyze sensation. In the twelfth-century in Celtic manuscript on materia medica mention is made of a draught which was used by the early Irish to induce sleep; and in the fifteenth century, on occasions of surgical operations, patients were put to sleep by means of that which was termed "The Sleeping Sponge." Reginald Scott, in the sixteenth century, wrote of an anaesthetic made of opium, mandragora bark, and henbane root; and Shakespeare's referenecs to "drowsy syrups" are proverbial. Opium as an anaesthetic—both by inhalation and by internal administratiton—is declared to have been used in the eighteenth century, and during the same period other means of producing insensibility were suggested.

It were a work of supererogation for me to remind this distinguished presence of the brilliant discoveries in chemistry which created a new epoch in the history of anaesthesia; first, the discovery of Priestly, which led to administering gases and vapors by inhalation; then followed the experiments of Beddoes; the researches of Humphrey Davy on nitrous oxide; the inhalation of sulphuric ether, by Woolcombe, of Plymouth; and the conclusion of Faraday, in 1818, that the vapor of sulphuric ether produced similar effects to those caused by nitrous oxide. All of these valuable discoveries are now as a tale oft told, as is also the fact that Professor Thompson, of Glasgow, amused his students by occasionally permitting them to inhale ether and nitrous oxide until they became unconscious and appeared to be insensible to pain.

Says a well-known writer upon the subject of anaesthesia: "It is extraordinary that among all the investigators who for so many years stood upon the very brink of a great discovery, no one ventured over the threshold."

That the practical understanding of anaesthesia finally came, and same in an unexpected, indirect way, if such knowledge may ever be called indirect, is known to all within the sound of my voice. It is my privilege and my pleasure today to memorialize the great discoverer of anaesthesia, both because of his valuable work, and because the United States, the State of Georgia, and our medical association may claim him as their own.

On the first day of November, 1815, Crawford W. Long was born in the State of Georgia, in the village of Danielsville, a place of such modest proportions as to merit the affirmation of Washington Irving when he aid: "Genius loves to bring forth her offsprings in by-corners. She seems to delight in disappointing the assiduities of art, and to glory in the vigor of chance productions. She scatters her seeds to the winds, and though some may perish among stony places, others struggle bravely up into sunshine."

The ancestry of the discoverer of anaesthesia was highly respectable. His paternal grandfather, Captain Samuel Long, of Pennsylvania, distinguished himself during the Revolutionary War; he was one of General Lafayette's officers at Yorktown, and saw the independence of his country triumphantly established. He moved to Georgia, and here his son, James Long, became a superior scholar, a profound student of the law, was for years a member of the Senate, and was regarded as one of the prominent men of the commonwealth. James Long was an intimate friend of Georgia's great statesman, William H. Crawford, and as a result of this friendly relation he gave to his first born son the name of Crawford.

If it were possible to penetrate the remote and occult sources of character and temperament as they are transmitted from one generation to another, perhaps we might trace the force and beauty which governed the life of Crawford W. Long, to the enduring impressions stamped upon his imagination by the sentiments of his distinguished parentage.

Be that as it may, he certainly had no cause to be ashamed of his ancestry. We have no superstitious veneration for that which is termed "blue blood," especially when it is the reproach of degenerate offspring, but we very properly rejoice with the man who can trace his descent from an honored line.

Crawford W. Long early displayed signs of unusual ability. His primary education was quickly accomplished, and he matriculated at Franklin College—now the University of Georgia—at a peculiarly early age, graduating from this institution when only nineteen, standing second in his class, and receiving the degree of Master of Arts. After studying for one year at the University of Pennsylvania, he was graduated from that renowned institution, where he had largely and successfully devoted time to experimental work. He then spent a year in New York, and while there attained reputation as a skillful surgeon.

In 1841, because of family importunities, Crawford W. Long returned to Georgia. He began the practice of medicine in the village of Jefferson, far from the bustle of the great world, remote from railroads and other necessities of modern life, truly a "nestling place for genius."

Dr. Long, albeit, yet a young man, soon acquired an extensive practice. His abilities were apparent. His quiet, thoughtful bearing attracted people to him. It may be declared that there was more in his *silence* than in the *words* of many men. Throughout life Dr. Long was one of those men whom, according to George Eliot, "we can best know by entering with them their homes, and hearing the voice with which they speak to the aged and young about their hearthstone, and witnessing their careful thought for the everyday wants of everyday companions," He bore a fine character, and "character," says Phiilips Brooks, "is like a bell which rings out sweet music, and which, when even accidentally touched, resounds with music."

It was apparent to both old and young that Crawford W. Long had come into the world to better his fellow creatures. His office became the place of sojourn of those who desired a pleasant evening, especially of the young men of the village. About that time the inhalation of laughing gas, as an exhilarant, was much discussed. Lecturers on chemistry would sometimes entertain by giving a "nitrous oxide party," during which the participants would become drunk from its inspiration. It was in the winter of 1841 that some young friends importuned Dr. Long to permit them to indulge this pastime in his office. The physician had no means of preparing nitrous oxide gas, but suggested that sulphuric ether would produce similar exhilaration. The ether was produced; the young men inhaled and became hilarious. During the period of mirth some of them received bruises. The young medical practitioner noted that these bruises were not accompanied with pain. In consequence he divined that ether must have the power of rendering one insensible to pain, and from this simple observation came the great discovery of anaesthesia.

Just here it may not be improper to remind ourselves that many of the brightest achievements of science are the results of slight observations, as the incident of Sir Isaac Newton and the falling apple proves. We are taught that the art of printing, probably the parent of more good than all others, owes its origin to rude impressions taken from letters carved on the bark of a beech tree—so trivial a matter that thousands would have passed it over with neglect. We are taught that the stupendous results of the steam engine may be traced to the chance observation of steam issuing from a bottle just emptied and placed casually near to a fire. We are also taught that electricity was discovered by some one noticing that a piece of rubbed glass attracted bits of paper. Every one now appreciates the importance of these wonders, yet they were the results of slight observations.

"Nothing is too little for the attention of man," says an old maxim upon the walls of the workshop of Peter the Great. The thoughtful subject of this paper found nothing in his profession too small for careful attention. He promptly determined to prove the value of his discovery, and during the month of March, 1842, ether was administered to Mr. James Venable until he was completely anaesthetized, then a small cystic tumor was taken from the back of his neck. To the amazement of the patient he experienced no pain, and surely this was complete anaesthesia From five to eight other cases, testing the anaesthetic power of ether, were satisfactorily dealt with by Dr. Long during the years 1842 and 1843—quite a goodly number when it is remembered that surgical operations were not frequent in the country practice of a young physician more than half a century ago.

Dr. Crawford Long's surgical operations, under ether, were exhibited to medical men and also to persons of the community, as established by affidavits of persons operated upon, and of witnesses to the operations. Says Ange De Laperriere, M. D., of Jackson County: "I do certify that the facts of Dr. C. W. Long using sulphuric ether by inhalation to prevent pain in surgical operations, was frequently spoken of and became notorious in the County of Jackson, Georgia, in the year 1843." In May, 1843, Drs. R. D. Moore and Joseph B. Carlton, for many years leading physicians in the city of Athens, Georgia, discussed the trial of Dr. C. W. Long's discovery in a case of surgery before them. They were unfortunately prevented from making the experiment by having none of the fluid at hand. Mrs. Emma Carlton, widow of Dr. Joseph B. Carlton, who died recently in Athens after living here for many years, signed the following: "I do certify that Dr. Crawford W. Long, of Jefferson, Jackson County, advised my husband, Dr. Joseph B. Carlton, a resident of Athens, Georgia, to try sulphuric ether as an anaesthetic in his practice. In November or December, 1844, in Jefferson, Georgia, while on a visit to that place, in the office of Dr. Long, my husband extracted a tooth from a boy who was under the influence, by inhalation, of sulphuric ether, without pain—the boy not knowing when it was done. I further certify that the fact of Dr. Long using sulphuric ether, by inhalation, to prevent pain, was frequently spoken of in the County of Jackson at this time, and was quite notorious."

It is to be regretted that Dr. Long did not *at once* make known to the world his great discovery of anaesthesia. Considered from a present point of view, his delay seems extraordinary. But it must not be forgotten that since that period the world has moved with exceeding rapidity. Sixty-five years ago, for a young medical practitioner in an obscure village, far from contact with centers of thought, removed from railroads, enjoying but modest postal facilities, with no great hospital organizations or medical associations to confirm his professional research, for a modest, diffident, young physician to claim so startling a discovery as anaesthesia has proven to be, without first securing most exhaustive proof of its worth, would have brought upon him the adverse criticism of his elders, and possibly the laughter of his colleagues.

Dr. Crawford Long as a young man, in his maturity, and when "nearing life's last white milestone," was ever a modest, unassuming gentleman. He sought no vain publicity. He fostered no extravagant aspirations. He was only a wise, patient, careful seeker after truth. He worked and waited, resolving to make the most comprehensive report of his discovery, after testing all kinds of cases. His great work was slowly stealing forth and beginning to perform its beneficient and beautiful office, but he, the author, was standing quietly back in the shadow. He was hoping much, but at the same time was ruling himself, thereby meeting the application of John Milton's words when he said: "He who ruleth himself is more than a king."

Had Dr. Crawford Long promptly made known the results of his experiment it would have assured the distinguished honors to which he was entitled, and would have prevented long-continued controversy as to who was really the discoverer of anaesthesia. A careful examination of the question clearly shows that two and a half years elapsed after the discovery by Crawford W. Long, before Dr. Wells, of Hartford, knew the anaesthetic power of nitrous oxide; that four and a half years passed after Dr. Long's initial experiment before Dr. Morton claimed to have the same knowledge. Morton is declared to have received the suggestion from Jackson; the latter claims to have made the discovery about the time Dr. Long made it, but left it to Morton to practically prove. Says Hugh H. Young, of Johns Hopkins Hospital, in his intersting pamphlet entitled, "Long, the Discoverer of Anaesthesia," "The immediate and universal *use* of anaesthesia in surgery is due to the great Boston surgeons, Warren, Hayward and Bigelow."

In 1849, Morton petitioned Congress for a reward as the discoverer, but he was opposed by the friends of Wells and Jackson The friends of Morton and Wells presented volumes of testimony to the Senate of the United States in behalf of their candidates, but Jackson afterwards acknowledged the justice of Dr. Long's cause. For five years Crawford W. Long refused to take any part in the controversy. Never, indeed, did he ask pecuniary reward, but he naturally desired to be recognized as the discoverer of anaesthesia, and to that effect wrote an article for the Boston Medical Journal.

Confronted by so formidable an opponent as Long, the friends of Morton and Wells finally seemed to lose hope, the bill before Congress was allowed to die, and it was never resurrected. In 1877, Dr. J. Morton Sims investigated the claims of Dr. Long to the discovery of anaesthesia, and was convinced of their merit. He demanded their recognition by the medical profession, Dr. Long especially desiring the endorsement of the American Medical Association. It was but a short time afterwards that Dr. Long died, on the 16th day of June, 1878, in the city of Athens, Georgia, for many years the place of his residence.

The "Eclectic Medical Association" soon passed a decree in favor of Long, as did a number of minor societies; and Dr. Henri Stuart, founder of the Woman's Hospital in New York, presented a portrait of the discoverer of anaesthesia to the University of Georgia. A report has been circulated that a statute to the honor of Dr. Long has been placed in the City of Paris, France, but I am not informed as to the accuracy of such report.

Georgia has all along recognized Dr. Crawford W. Long as the discoverer of anaesthesia, and when Governor Alexander H. Stephens was requested to name two great Georgians whose portraits might hang in the National Gallery, he designated Oglethorpe and Long. Thus Georgia has recognized her distinguished son, but Georgia has been slow, very slow, in paying *all* of the tribute due her renowned dead, for the memory of this son has not yet been perpetuated in marble or bronze. The village of his birth, the other village which was the scene of his discovery, the town of his long residence and now custodian of his remains, the State Medical Association, the State University, the State herself, have yet failed to erect a public memorial to Crawford W. Long. The neglect has been unfortunate, and it should be quickly remedied.

To preserve the memory of thtose who have conferred great benefits, is both a privilege and a duty. To honor the illustrious dead is to stimulate the living to higher ideals and loftier ambitions. It is a usage sanctioned by the wisdom of many ages of civilization. A Southern orator has said: "The city of ancient Athens was full of the memorials of actual history. Every street and square from the Piraeus to the Acropolis were adorned with statues of great men of the commonwealth, and twenty-one centuries have not extinguished this sentiment of veneration for the illustrious dead. Memorials of such men are to be found in every civilized land. On the banks of the Danube there stands a noble marble structure, called the Hall of Heroes, filled with effigies of the great sons of Germany. By the soft blue waters of Lake Lucerne stands the Chapel of William Tell. In the black aisle of the old cathedral at Innspruck, the peasant kneels before the statue of Andreas Hofer. In her senate hall England bids her sculptors still to place the images of her noblest sons. Two hundred years after the death of Shakespeare a monument is erected to honor him, though his own works had already immortalized the name. Even now plans are being made for erecting a building in Washington City to memorialize Thomas Jefferson. The memory of Dr. Benjamin Rush is perpetuated in stone; and everywhere we may find similar tributes to the great men of various callings. In the City of Washington rises a monument to the Father of his Country—this great American republic of ours.

Gentlemen of our Georgia Medical Association, let us not defraud *our* illustrious deed of their rightful memorials. Let us wait no longer to proclaim by noble, beautiful and enduring art, this one of our number who gave an unsurpassed gift to his profession and to the world.

We are to be congratulated that some have not been so unmindful as we, concerning this obligation, for a gentle reminder of our duty has quite recently come to us from that distinguished body, the State Federation of Women's Clubs—an organization that today a most potent factor for good in things educational, industrial and beautiful.

The Athens Chapter of the Federation of Women's Clubs has gladly undertaken the task of collecting an amount sufficient to erect a monument in honor of Dr. Long. This monument is to be at Athens, where repose the remains of the great discoverer, and will be erected in the name of the Medical Fraternity of Georgia. It is the earnest desire of those interested in this admirable undertaking to have a monument ready for unveiling during the gathering of our Medical Association at Athens in 1909.

Let us not prove forgetful of our interest in this memorial. In conformity with the usages sanctioned by ages, in conformity with the custom of our own time and our own country, in conformity with loving remembrance for our distinguished dead, let us unite our energies with those who are cheerfully and happily praparing to perpetuate in marble or bronze the memory of Crawford W. Long, the great discoverer of anaesthesia. Then may we exclaim with the poet:

"Patriots have toiled and in their country's cause
Died nobly. And their deeds, as they deserve
Receive proud recompense. We give in charge
Their name to the sweet lyre. The historic muse,
Proud of the treasure, marches with it down
To latest times; and sculpture in her turn
Gives bond in stone and ever-during brass
To guard them and immortalize her trust."

